# Structure-Based Virtual Ligand Screening on the XRCC4/DNA Ligase IV Interface

**DOI:** 10.1038/srep22878

**Published:** 2016-03-11

**Authors:** Grégory Menchon, Oriane Bombarde, Mansi Trivedi, Aurélie Négrel, Cyril Inard, Brigitte Giudetti, Michel Baltas, Alain Milon, Mauro Modesti, Georges Czaplicki, Patrick Calsou

**Affiliations:** 1 Institut de Pharmacologie et de Biologie Structurale, Université de Toulouse, CNRS, UPS, France; 2 Equipe labellisée Ligue Nationale Contre le Cancer, France; 3 Synthèse et physico-chimie de molécules d’intérêt biologique, Université de Toulouse, CNRS, France; 4 Centre de Recherche en Cancérologie de Marseille, CNRS, UMR7258, Marseille, F-13009, France; 5 INSERM, U1068, Marseille, F-13009, France; 6 Institut Paoli-Calmettes, Marseille, F-13009, France; 7 Aix-Marseille Université, Marseille, F-13284, France

## Abstract

The association of DNA Ligase IV (Lig4) with XRCC4 is essential for repair of DNA double-strand breaks (DSBs) by Non-homologous end-joining (NHEJ) in humans. DSBs cytotoxicity is largely exploited in anticancer therapy. Thus, NHEJ is an attractive target for strategies aimed at increasing the sensitivity of tumors to clastogenic anticancer treatments. However the high affinity of the XRCC4/Lig4 interaction and the extended protein-protein interface make drug screening on this target particularly challenging. Here, we conducted a pioneering study aimed at interfering with XRCC4/Lig4 assembly. By Molecular Dynamics simulation using the crystal structure of the complex, we first delineated the Lig4 clamp domain as a limited suitable target. Then, we performed *in silico* screening of ~95,000 filtered molecules on this Lig4 subdomain. Hits were evaluated by Differential Scanning Fluorimetry, Saturation Transfer Difference - NMR spectroscopy and interaction assays with purified recombinant proteins. In this way we identified the first molecule able to prevent Lig4 binding to XRCC4 *in vitro*. This compound has a unique tripartite interaction with the Lig4 clamp domain that suggests a starting chemotype for rational design of analogous molecules with improved affinity.

DNA double strand breaks (DSBs) are the main lesions responsible for cell death after treatment with ionizing radiation (IR) or radiomimetic molecules^1^. As a result DSB cytotoxicity is widely exploited in anticancer treatments by radiotherapy or chemotherapy with clastogenic agents. The ability of cells to repair DSBs in their genome largely accounts for the variable cytotoxic effect of these agents^2^. Non-Homologous End-Joining (NHEJ) is the predominant DNA repair mechanism by which cancer cells resist radio and radio-mimetic chemotherapies. Unlike Homologous Recombination that repairs DSBs in a template-dependent manner, NHEJ ligates the break *in situ.* In addition NHEJ operates in all phases of the cell cycle^3^. NHEJ is thus a good target for strategies aimed at increasing the sensitivity of tumors to clastogenic anticancer treatments^4^.

Briefly, two main complexes operate during NHEJ: 1/ DNA-dependent protein kinase (DNA-PK) recognizes, protects and brings DSB ends closer and, *via* its auto-phosphorylation, promotes loading and activation of processing factors necessary to clean the ends before ligation; 2/ the Cernunnos-XLF/XRCC4/DNA ligase IV complex is responsible for the final ligation step^5^,^6^,^7^,^8^. Inside this ligation complex, XRCC4 directly interacts with DNA ligase IV (Lig4) and stimulates break rejoining catalysis^9^,^10^,^11^. Lig4 is unstable in the absence of XRCC4^12^.

To date, the most common target in NHEJ inhibiting strategies has been the serine-threonine kinase activity of DNA-PK^4^,^13^. The ligation complex is also an appealing target since Lig4 defective cells are particularly radiosensitive^14^, but it has not been as greatly exploited. Cross-inhibition of Lig1 and Lig3 human DNA ligases by Lig4 catalytic inhibitors has been observed due to the highly conserved catalytic mechanism^15^. Targeting XRCC4/Lig4 interface would avoid this drawback. A loss of Lig4 is observed after destabilization of its interaction with XRCC4^16^. Moreover, Lig4 has a non-catalytic function in NHEJ as a regulator of DNA-PK autophosphorylation that controls DNA-ends access to processing enzymes^17^. Hence targeting the XRCC4/Lig4 assembly could produce a double effect through both inhibition of NHEJ and of rescuing DSB repair pathways by blockage of DNA-PK at DNA ends^17^,^18^. Up to now, a few Lig4 catalytic inhibitors have been isolated but all cross-inhibit to various extents at least one of the two other human DNA ligases^19^,^20^. No molecule able to interfere with the assembly of XRCC4 and Lig4 has been reported.

The active form of the XRCC4/Lig4 complex is composed of one XRCC4 homodimer and one Lig4 monomer^21^,^22^ (Fig. 1A). Lig4 differs from the other human DNA ligases by the presence of two tandem BRCA1 (breast cancer associated) C-terminal (BRCT) domains at its C-terminus (amino acids 654–911). Lig4 catalytic core is connected to the C-terminus with a flexible linker^23^,^24^. XRCC4 consists of 336 amino acids and bears a globular head at the N-terminus and a coiled-coil tail at the C-terminus (C-ter) that mediates dimerization and interaction with Lig4. Structural and functional studies have shown that Lig4 interacts with XRCC4 through an extended region comprising the linker between the two Lig4 BRCT domains (β-hairpin region (amino acids 759–770) and Helix1-Loop-Helix2 clamp domain (amino acids 771–803), both forming a minimal XRCC4 interacting region – thereafter named XIR) followed by a portion of the second Lig4 BRCT domain (BRCT2)^16^,^22^,^25^ (Fig. 1A,B).

XRCC4/Lig4 interface spans over ~2900 Å^2^,^16^. The XRCC4/Lig4 interaction resists high salt or urea^9^,^10^,^22^ and organic solvents^26^. Indeed, the binding constant between the Lig4 XIR fragment and XRCC4 is in the nanomolar range^21^,^26^ likely precluding disruption of the preformed complex by small molecules. Nevertheless, interference with the assembly between XRCC4 and Lig4 in human cells has proven to be a feasible radio-sensitization strategy through over-expression of XRCC4^27^ or Lig4 fragments (our group,^16^). The catalytic domain of Lig4 is not expected to impact the Lig4-XRCC4 interaction^23^,^24^. Therefore, we conducted an exploratory study aimed at identifying molecules able to interfere with XRCC4/Lig4 assembly by *in silico* screening on the XRCC4-interacting domain of Lig4 (Lig4 clamp domain) accompanied by biophysical and biochemical selection of hit molecules.

Here, we report the isolation of the first compound able to target the Lig4 clamp domain and to prevent the interaction of the clamp-containing Lig4 C-ter domain with XRCC4 *in vitro*. This result (i) validates the minimal clamp domain of Lig4 as a target for *in silico* screening strategy, (ii) supports the feasibility of preventing the extremely high-affinity XRCC4/Lig4 interaction with small molecules *in vitro*, (iii) suggests a starting point model for the rational design of stronger inhibitors of this challenging protein-protein interaction.

## Results

### Molecular Dynamic simulation of the XRCC4/ Lig4 C-ter complex

**Figure 1:**
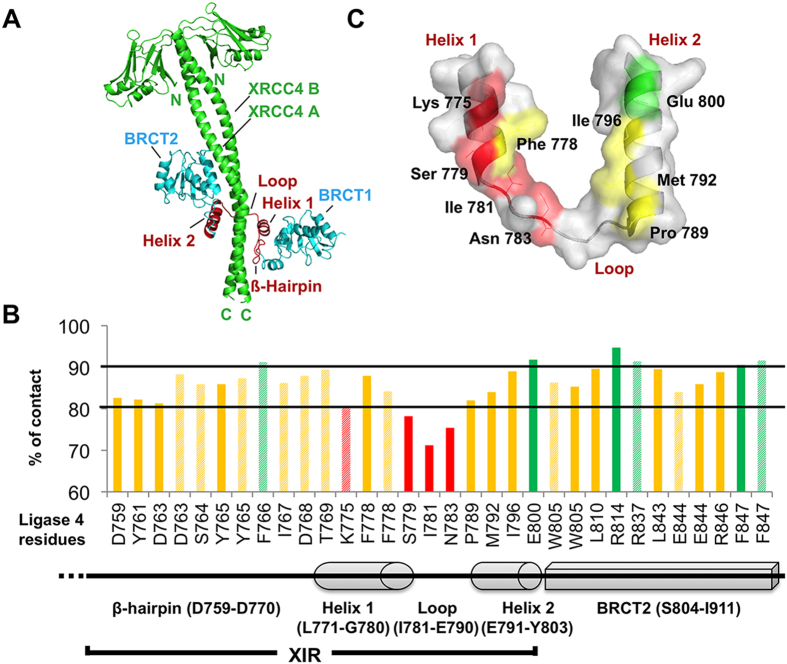
Structure of the human XRCC4/Lig4 complex and analysis of persistent contacts by Molecular Dynamics Simulation. (**A**) Crystal structure of the XRCC4/C-ter Lig4 complex (PDB entry 3II6) : the clamp domain (helix1-loop-helix2) is in red. (**B**) Distribution within the Lig4 C-ter domain of persistent contacts with XRCC4. Dashed and full boxes correspond to residues contacting XRCC4 A and B chains, respectively. Green, yellow and red colors highlight very persistent (contact >90%), persistent (80%< contact <90%) and poorly persistent (contact <80%) residues, respectively. (**C**) Cartoon and surface representation of residues within the Lig4 clamp domain according to persistent contacts with XRCC4. The colors correspond to residues as in B.

To get quantitative insights into the dynamics of critical residues involved in the interaction between XRCC4 and the Lig 4 C-ter domain (residues 654–911), we performed a 10 ns Molecular Dynamics (MD) simulation on this complex. From this run, we analyzed the evolution of the root mean square deviation (RMSD) of the atomic coordinates for both interacting proteins as well as persistent intermolecular contacts, defined as the distances between protein/ligand heavy atoms, which do not exceed 3 Å and exist in at least 80% of all trajectory frames. As a starting model, we used the crystallographic structure of the complex (PDB entry 3II6) (Fig. 1A). The solvated system with both proteins equilibrated after around 3 ns and RMSD was stable until the end of the simulation. The results have been corrected for the overall rotation and translation of the whole complex. Frames corresponding to the remaining 7 ns stable trajectory were then extracted to analyze persistent intermolecular contacts between the Lig4 C-ter fragment and XRCC4 A and B chains with in-house software (Fig. 1B,C). In this analysis, a contact between two residues was judged as persistent if it reached a threshold value of 80% and very persistent with a value of 90% over the whole 7 ns trajectory. The histogram of all the XRCC4 interacting residues of the Lig4 C-ter domain with their percentage of contact provided details on the most critical intermolecular interactions involved in complex stability (Fig. 1B). According to the 90% threshold value, Phe766 (β-Hairpin), Glu800 (Helix2), Arg814, Arg837 and Phe847 (BRCT2 domain) were contacts predicted as the most energetically favorable hotspots in the XRCC4/ Lig4 C-ter association (Fig. 1B). The fact that several of those persistent contacts were present in the BRCT2 domain (S804-I911) was in line with data reporting that the BRCT2 domain is critical to stabilize the Lig4 C-ter structure on XRCC4^16^. However, additional studies using ectopic expression of truncated Lig4 constructs have also shown that the sole BRCT2 domain was not sufficient to prevent formation of the endogenous XRCC4/Lig4 complex^16^. Moreover, a recent biophysical study with fragments from the XRCC4 Interacting Region (XIR) reported that a competitive displacement of the XIR from XRCC4 was only significant with either fragments of Lig4 containing Helix2 or with the Helix2 alone^26^. Accordingly, our MD simulation revealed that in addition to the BRCT2 domain, the small Lig4 Helix2 fragment had also several strong contacts while only Phe778 in Helix1 reached a percentage of contact over 80% (Fig. 1B,C). Thus, both biophysical data and our MD simulations highlight the importance of this region of Lig4. Considering that the clamp part of Lig4 (Helix1-Loop-Helix2; residues 771–803) was essential for interaction with XRCC4^16^,^25^ and that its shape (defining a cavity) might be more suitable for molecular docking than other XRCC4-interacting portions of Lig4 (Fig. 1C), it was then used as a structural basis for virtual screening analysis.

### Virtual screening on the Lig4 clamp domain

**Figure 2:**
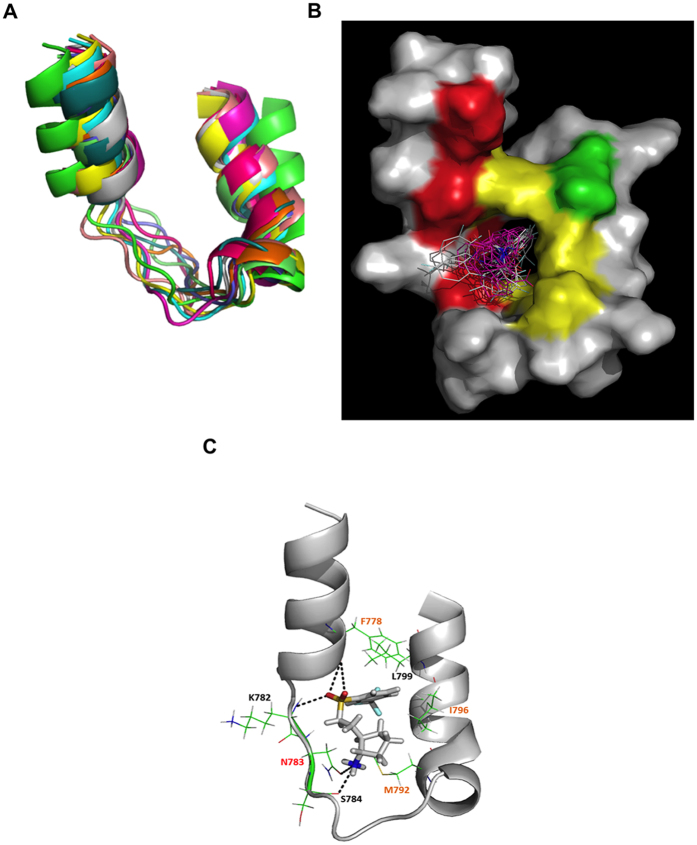
Virtual screening on the Lig4 clamp domain. (**A**) Overlap of the eleven MD simulation-extracted structures of Lig4 clamp domain used for molecular docking. (**B**) Example of clustering analysis within a Lig4 clamp structure after docking, showing molecule #93291 with the superimposition of ten conformers in a single position cluster. The clamp structure is represented in surface with persistent contact colored as in Fig. 1C. (**C**) Example of a lowest-energy conformer for a Lig4 clamp interacting compound (compound #231) from the docking step. The clamp is represented in cartoon and residues making contacts with the molecule # 231 are highlighted and colored as in Fig. 1C. H-bonds are represented in black dashed lines.

The main limitation during the docking process is the docking scoring function, which evaluates and attributes a score to a ligand pose inside a receptor, and the fact that all the ligand and receptor conformations are not accessible. This usually leads to a very low hit rate during the virtual screening process, with a high number of both false positives and negatives. To improve the hit rate, we chose the Relaxed Complex Scheme (RCS) method in which docking is performed on an ensemble of receptor conformations extracted from a long MD simulation run^28^. This allows accounting for protein flexibility during the screening process and it has been applied successfully in many examples^29^,^30^,^31^. The final goal of our targeting strategy on the XRCC4/Lig4 complex was not to disrupt their very high affinity interaction but rather to prevent their initial association. Therefore we needed to establish the structure of the Lig4 clamp domain separated from XRCC4. Since the C-terminal structure of the Lig4 was only available with its XRCC4 partner (PDB entry 3II6), we first virtually assessed its intrinsic flexibility when present alone by running a 15ns MD simulation in a periodic box with explicit solvent. To simulate different clamp conformations, we kept both BRCT domains to account for structural constraints. After 3 ns, RMSD for the clamp domain remained stable. After equilibration, Helix1 and Helix2 became closer mainly by a hydrophobic contact between F778 from Helix1 and L799 from Helix2 (data not shown). This new link created a large cavity inside the tripartite structure, whose surface varied with the simulation and the side chains flexibility but which was very well suited to accommodate inhibitor molecules (Fig. 2A,B). We set the cluster radius to 2 Å and extracted eleven major main chain conformers for subsequent docking. These clamp structures thus likely represented the most probable conformations of Lig4 in solution when not in complex with XRCC4 (Fig. 2A). This conformationally sampled Lig4 clamp receptor was then used to dock a filtered library of around ~95,000 molecules. These molecules were provided by a subset of the ZINC chemical database (“Clean Drug-Like” subset)^32^, further enriched by an in-house library. The initial set of molecules collection was first filtered according to specific physico-chemical properties (molecular weight <450 Da; cLogP and Solubility Forecast Index (SFI)^33^ between −3 and 1; polar surface area <110 Å^2^; H-bond donors and acceptors between 0 and 5; rotatable bonds between 0 and 6 and charges between −1 and 1). In addition, duplicates and known toxic or false-positive molecules were removed. Filtered molecules were then submitted to a pre-screening step by rigid docking on each of the eleven independent clamp structures using Autodock Vina^34^ as described in Materials and Methods. BRCT domains were removed and the grid boxes for docking were centered on the clamp structures. This step allowed the ligands to be fully flexible and the clamp was kept rigid to allow a receptor conformation selection by the ligands. Flexibility for the whole protein/ligand complexes was then re-introduced during the next MD simulation step. After the docking, each molecule was ranked according to its Vina score^34^, its clustering and its interactions with residues making persistent contacts with XRCC4. Those with a score ≥−7 kcal/mol, making at least one polar and one hydrophobic contact within the clamp and having only one cluster were chosen for the next step, with each lowest energy conformer being selected as representative. As examples, several conformers of a molecule (#93291) in a single position cluster are shown in Fig. 2B. This molecule reached the Vina score of −7 kcal/mol, fitted perfectly inside the clamp cavity and contacted residues from the Helix1, loop and Helix2. Another example of a molecule (#231) with a good binding score is depicted in Fig. 2C, showing binding mode details between the compound and the clamp. In this example, the stability of the molecule was due to hydrogen bonds network between the sulfur dioxide and the amide group of the K782 main chain and between the ammonia and the carbonyl groups of the N783 side chain and S784 main chain. Moreover, the ligand accommodated in a hydrophobic cleft formed by M792, I796, F778 and L799 side chains. The residues that mostly contributed to the best ligands interaction were previously identified as critical residues from the MD simulation of the XRCC4/ Lig4 C-ter complex: especially I796 and M792 from Helix2, N783 from the loop and F778 from Helix1. These general features were further used to carefully discriminate between potential hits.

**Figure 3:**
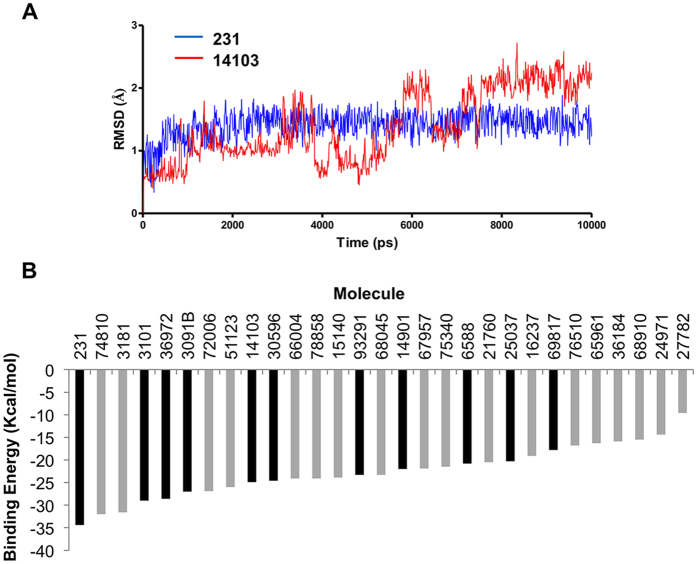
Examples of RMSD graphs and binding energy histogram of selected molecules. (**A**) RMSD evolution with time for a stable molecule (#231, blue curve, RMSD score = 3) and for an unstable one (#14103, red curve, RMSD score = 1) over a 10 ns MD simulation and within the clamp domain of the Lig4 C-ter fragment. (**B**) Compounds selected after docking onto the Lig4 clamp domain were ranked according to binding energy. Black and gray boxes correspond to compounds making or not persistent contacts with the target throughout the MD simulation, respectively.

From this initial docking step, we found 29 potential clamp interacting compounds that were subsequently submitted to a 10 ns MD simulation on the full Lig4 C-ter structure. The docking is a suitable tool to determine the positioning of a ligand into a binding site but cannot be used to predict binding energies and rank candidates accordingly^35^. MD simulations of each of the target/molecule complexes introduce a dynamic view of the interactions in explicit solvent, including protein side chains movements and adaptation to the ligands. The sampling of different stable protein and ligand conformations generally gives a more accurate prediction of the binding energy^36^. Thus, we determined the stability over time (RMSD) of the interaction, persistent or water-mediated contacts for each molecule inside the clamp domain as well as details on the molecule binding mode and energy. A RMSD score (from 1 to 3) was attributed to each molecule according to mobility inside the clamp domain (Table 1 and Fig. 3A for an example). A contact between a clamp residue and a molecule was judged persistent if it was present during 90% of the time within the trajectory (Fig. 3B). Eleven molecules (hits) were found to be highly stable within the binding site. These hits were retained according to RMSD and persistent contacts analyses and classified according to their binding energy (Table 1).

### Biophysical validation of molecules binding to the Lig4 C-ter domain

**Figure 4:**
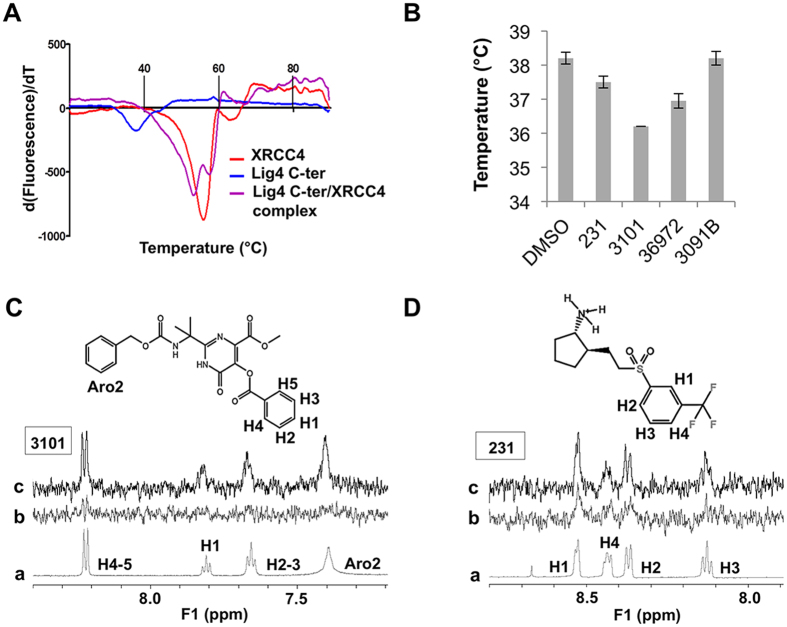
Thermal stability analysis for the Lig4 C-ter domain and XRCC4 constructs alone or in complex by Differential Scanning Fluorimetry (DSF) and analysis of the interaction of selected compounds with the Lig4-Cter domain by DSF and Saturation Transfer Difference -NMR spectroscopy. (**A**). Unfolding was followed with 10X SYPRO Orange dye and between 20 and 89 °C. Fluorescence signal derivative was plotted against the temperature and Tm values were estimated from the transition midpoint of the fluorescence curves. (**B**) Analyis by DSF of the thermal stability of the Lig4 C-ter domain in the presence of selected compounds or DMSO, as indicated. Unfolding is followed with 10X SYPRO Orange dye and between 20 and 89 °C. Tm values are estimated from the transition midpoint of the fluorescence curve (mean ± SD, n = 3). (**C**,**D**) One-dimensional ^1^H (a) and ^1^H STD-NMR spectra of the two molecules 3101 and 231 alone (b) or in association with the Lig4 C-ter fragment (c). Protons making close contacts with the protein show significant resonance intensity enhancements. Due to baseline distortion and buffer, only the aromatic regions are represented. Each corresponding proton is assigned on the spectrum and labeled on the molecule.

To go further in the screening process, we investigated the compound-protein interaction by two methods: Differential Scanning Fluorimetry (DSF)^37^ and Saturation Transfer Difference (STD) - NMR spectroscopy. These experiments are complementary biophysical approaches to determine binding. STD-NMR is particularly well adapted to the detection of weak affinity ligands in condition of large excess compared to the protein and in a fast exchange regime on the NMR timescale^38^. Due to availability and solubility requirements, only four molecules (#231, #3101, #36972 and #3091B) were integrated in our tests. XRCC4 (residues 1–336) and the Lig4 C-ter fragment containing both the clamp and BRCTs domains (residues 654–911) were expressed and purified as described in Materials and Methods. The Lig4 C-ter recombinant fragment had a melting temperature close to 38.2 °C in the buffer conditions used. The Tm was low in comparison with XRCC4 protein (Fig. 4A), emphasizing the intrinsic instability of this Lig4 fragment when not in complex (unpublished data). Except for #3091B, all the compounds altered the Lig4 C-ter thermal stability by decreasing the melting temperature as compared with the DMSO control. Notably, the molecule #3101 decreased the thermal stability by 2 °C (Fig. 4B, Table 2). A decrease in Tm has been attributed to ligands that destabilize proteins by binding primarily to the unfolded state of the protein^39^. MD on the Lig4 C-ter fragment with of without #3101 revealed that ligand binding to the clamp increases the internal dynamics of the BRCT1 domain, possibly contributing to the observed decrease in Tm (data not shown). STD-NMR was performed in 71-fold molar excess conditions of ligand over the Lig4 C-ter target. As judged by significant resonance intensity enhancements in the molecule aromatic regions and by comparison to control experiments without protein, saturation transfer efficiently occurred only with the molecules #3101 and #231 compared to control experiments without protein (Fig. 4D and Table 2). The importance of using several biophysical techniques in combination is underlined with the compound #36972 which, in spite of inducing a significant decrease in protein thermal shift (Fig. 4B), showed no binding in STD-NMR. In contrast, both biophysical methods clearly established binding to the Lig4 C-ter fragment of both molecules #3101 and #231 which were further evaluated in interaction assays with purified recombinant proteins.

### Evaluation of candidate molecules in an XRCC4/ Lig4 C-ter interaction assay

**Figure 5:**
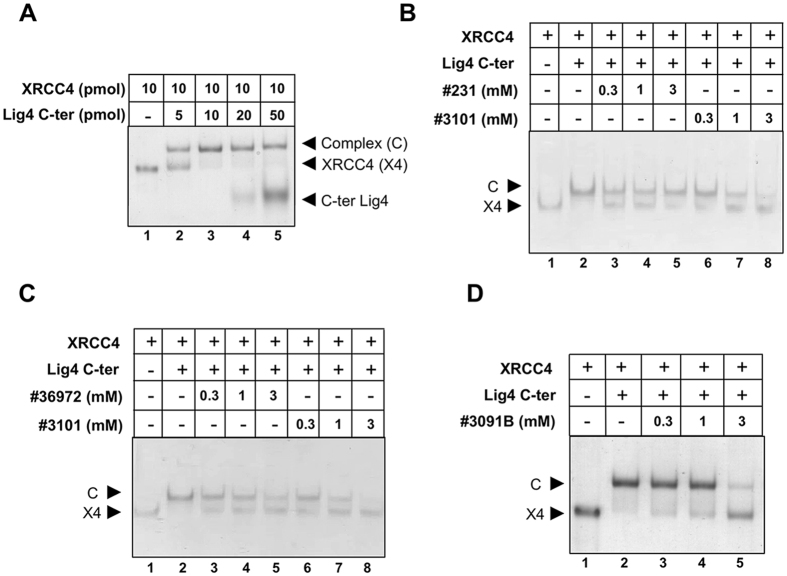
Functional analysis of selected molecules. (**A**) Native gel-based assay for the interaction between XRCC4 and the Lig4 C-ter fragment at the indicated concentrations. (**B,C**) Analysis of the inhibition activity of selected molecules at the indicated concentrations in the interaction gel-based assay. (**D**) Analysis of the inhibition activity of molecule 3091B at the indicated concentrations in the interaction gel-based assay. The arrows indicate the positions of XRCC4 protein (X4) and of its complex with the Lig4 C-ter fragment (**C**).

First, interaction between purified recombinant XRCC4 protein and Lig4 C-ter fragment was tested by DSF. Individual proteins showed DSF curves with a transition profile characteristic of folded proteins and when mixed, exhibited an increase in their respective thermal stability, indicative of their ability to form a complex (Fig. 4A). The effect of our candidates on the formation of the XRCC4/ Lig4 C-ter complex was further assessed in a native gel-based interaction assay. Purified XRCC4 was incubated with increasing amounts of purified Lig4 C-ter domain and the reaction mixture was run on a native gel. At equimolar concentrations, both components were fully engaged in complex formation, thus confirming their functionality (Fig. 5A). In the interaction buffer used, molecules #231, #3101, #36972 and #3091B were shown to be soluble up to 3 mM by NMR. Each candidate inhibitor was first pre-incubated with Lig4 C-ter and XRCC4 was then added in the mixture. The activity of each compound was assessed as the extent of disappearance of the XRCC4/ Lig4 C-ter complex. As shown in Fig. 5B,C, only molecule #3101 had a dose-dependent inhibitory effect on the XRCC4/ Lig4 C-ter association. Notably, when #3101 was pre-incubated with XRCC4 instead of Lig4 C-ter, no inhibitory activity was found on XRCC4-Lig4 C-ter interaction (data not shown), arguing against promiscuity linked to compound aggregation. Molecule #3091B was the nearest synthesis precursor of compound #3101 (Fig. S1) and displayed an inhibitory effect only at the highest concentration used (Fig. 5D). Other compounds derived from the same dihydroxypyrimidine carboxamide scaffold were synthetized (Fig. S1). None exhibit improved activity compared to compound #3101 (data not shown).

### Specificity of #3101 binding to Lig4 C-ter

**Figure 6:**
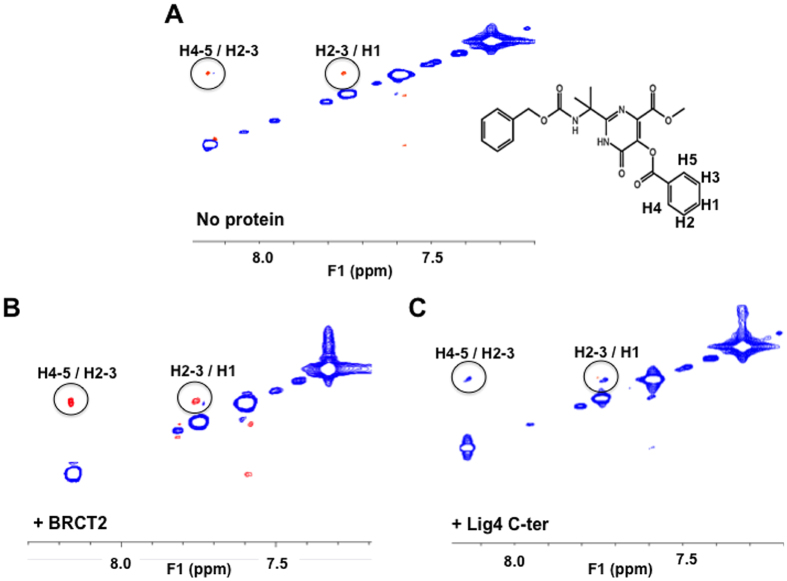
Analysis of the interaction of compound #3101 with the Lig4 C-ter and BRCT2 domains by TR-NOESY NMR spectroscopy. (**A**) TR-NOESY spectrum (mixing time 200 ms) of #3101 (1 mM concentration) alone in NMR buffer. The crosspeaks (in circles) showing spatial proximity between H4-5 and H2-3 and between H2-3 and H1 are due to positive NOEs (red, sign opposite diagonal peaks). (**B**) TR-NOESY spectrum (mixing time 200 ms) of #3101 (1 mM) with 14 μM of BRCT2 domain and in NMR buffer. The crosspeaks (in circles) showing spatial proximity between H4-5 and H2-3 and between H2-3 and H1 are due to positive NOEs (red). (**C**) TR-NOESY spectrum (mixing time 200ms) of #3101 (1 mM) with 14 μM of Lig4 C-ter domain and in NMR buffer. The crosspeaks (in circles) showing spatial proximity between H4-5 and H2-3 and between H2-3 and H1 are due to negative NOEs (blue, same sign as diagonal peaks).

Several features argue for the specificity of #3101 binding to its intended target on Lig4 C-ter. First, TR-NOESY spectra were carried out for the ligand only and in the presence of purified Lig4 C-ter or BRCT2 domains (Fig. 6). When alone in the protein buffer, #3101 showed positive NOESY crosspeaks, characteristic of a non-aggregating small molecular weight compound (Fig. 6A). While positive NOESY crosspeaks were also observed for this ligand in the presence of the Lig4 BRCT2 domain, negative NOESY crosspeaks were observed in the presence of the Lig4 C-ter domain, altogether indicating an effective binding to the whole Lig4 C-ter and no unspecific binding to its BRCT2 subdomain (Fig. 6B,C). Moreover, saturation transfer efficiently occurred with the molecule #3101 in the presence of the Lig4 C-ter domain and again not the BRCT2 subdomain alone (Fig. S2A,B).

### A model of interaction between molecule #3101 and the Lig4 C-ter domain

**Figure 7:**
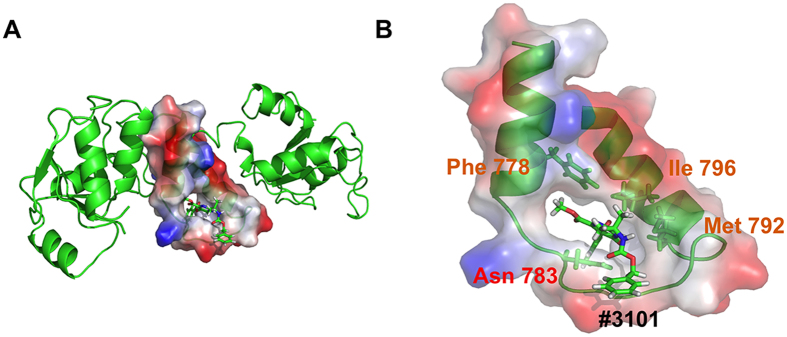
Interaction with the Lig4 C-ter domain analyzed by Molecular Dynamics Simulation. (**A**) Representation of the last frame from a 10 ns MD simulation between the Lig4 C-ter domain and molecule #3101. The clamp domain is represented in red/blue charge-smoothed surface (negative = red; positive = blue). (**B**) Close up view of the #3101 compound binding mode inside the Lig4 clamp domain with contacted residues highlighted and colored as in Fig. 1C.

The last frame of a 10 ns MD simulation between molecule #3101 and the Lig4 C-ter domain was extracted and analyzed for its binding mode (Fig. 7A,B). Although the simulation was performed with the Lig4 C-ter fragment comprising the two BRCT domains in addition to the clamp, the molecule remained localized within the clamp domain during the entire trajectory, confirming its binding specificity for this Lig4 domain. Notably, it exhibited a unique mode of binding to each of the three components of the clamp domain, establishing a persistent H-bond contact between its carbonyl group and amino group of the N783 side chain and hydrophobic interactions between its di-methyl and methoxy groups and the critical F778 and I796 side chains from helix1 and 2 respectively. This unique binding mode of compound #3101 provides a model for a Lig4 clamp-interacting ligand.

## Discussion

These results suggest a novel strategy for inhibiting DSB repair through interference with the XRCC4/Lig4 protein assembly, an interaction essential for DNA-end ligation by NHEJ. Using computational techniques together with biophysical and biochemical assays, we have discovered the first molecule targeting the Lig4 C-terminal domain and capable of preventing the binding of this domain to XRCC4 *in vitro*.

Starting with the 3D structure of the Lig4 C-ter domain at high resolution^16^, we performed a computer-aided screening campaign using a structure-based strategy. Computational identification of protein-protein interaction inhibitors has previously led to a number of successes^35^,^40^,^41^,^42^,^43^,^44^. The XRCC4/Lig4 interface extends from the beta-hairpin to the BRCT2 domains of Lig4 and covers about 2900 Å^2^ (Fig. 1A). This extensive interface was thus very challenging for docking studies and it was necessary to rationally define a more limited region as a potential target. Analysis of persistent contacts using Molecular Dynamics simulations on the XRCC4/Lig4 C-ter complex clearly identified the clamp Helix2 as a region particularly rich in energetically favorable hotspots in this complex. Subsequent conformational sampling analysis revealed that the Helix1 and Helix2 in the Lig4 clamp could form a cavity suitable for binding small molecules. This is consistent with biophysical studies from Junop’s group showing that the clamp Lig4 domain is able to efficiently prevent association between XRCC4 and the large Lig4 XIR in competition assays^26^. Therefore, we carried out a large virtual screening campaign specifically targeted on this clamp domain (Helix1-Loop-Helix2) of the Lig4 C-ter region.

We used AutoDock Vina^34^ as docking program and the Relaxed Complex Scheme (RCS) method^28^ to account for protein flexibility during the screening of potential inhibitors. RCS relies on molecules docking on several protein conformations computationally extracted by MD simulation. RCS has previously proven successful in several attempts to predict disruption of protein-protein interaction by small molecules^29^,^30^,^31^,^45^. Here, we carried out a Lig4 C-ter protein conformational sampling and a subsequent virtual screening campaign of ~95.000 filtered molecules on 11 clamp domain conformers. From a first set of 29 compounds selected according by a Vina score (≥7 kcal/mole) and clustering around critical XRCC4 interacting residues, only 11 were finally conserved according to persistent contacts analysis by MD simulations on the full Lig4 C-ter domain. Thus, coupling docking with MD simulations of protein/hit complexes clearly allows a more robust evaluation and ranking of promising hits.

Among the 11 selected hits, biophysical methods (DSF and STD-NMR) confirmed that compounds #231 and #3101 interact with the Lig4 C-ter region, but only compound #3101 was found to significantly prevent assembly of the XRCC4/ Lig4 C-ter complex in a native gel-based assay. In addition, data from STD-NMR and TR-NOESY experiments argued for the specificity of #3101 binding with Lig4 C-ter region. The tripartite binding mode of #3101 to all the three subdomains of the Lig4 clamp fragment may account for its inhibitory effect on the interaction between XRCC4 and the Lig4 C-ter domain. In agreement with this, MD shows that molecule #3101 tends to interact with clamp residues that establish the most persistent contacts with XRCC4. Interestingly, compound #3091B, a synthesis precursor of #3101 (Fig. S1), although selected in our docking campaign, was negative in biophysical assays and had a less pronounced inhibitory effect in protein-protein interaction assays. This emphasizes the importance of the additional terminal benzoyl group in molecule #3101 and supports the hypothesis that a larger molecule is more adapted to bind the large clamp domain surface^26^. In addition, other synthetized compounds of this family (Fig. S1) were less active than compound #3101. In that respect, structure activity efforts should now focus on the modification of the pyrimidine nitrogen group or the replacement of the carboxamide moiety in relation to docking experiments in order to obtain more active ligands from this scaffold.

Since Lig4 defective cells are exquisitely radiosensitive^14^, the XRCC4/Lig4 ligation complex is particularly appealing for a radio-sensitization strategy directed against NHEJ^4^. Lig4 is the unique ligase associated with this pathway with no other known function, making it an attractive target. Structural conservation among human DNA ligases^15^ is a major limitation of therapeutic approaches that target Lig4 catalytic activity. Indeed, known Lig4 inhibitors are promiscuous DNA ligase antagonists to various extents^19^,^20^. Impairing the XRCC4/Lig4 interaction demonstrated here might be expected to circumvent this drawback. Although the C-terminal region of XRCC4 may have a function in the complex with Lig4^46^, we believe that preventing Lig4 interaction with XRCC4 could be sufficient to inhibit ligation in cells, as demonstrated by the radiosensitizing effect of Lig4 fragments overproduced in cells^16^. Interestingly, specifically targeting the XRCC4/Lig4 interface may also have an additional application in gene editing which was recently shown to benefit from Lig4 inhibition^47^,^48^. From the present data, we can anticipate that radiosensitization or gene-editing applications would require compounds with very high affinity for the XRCC4-interacting region of Lig4. It is also conceivable that in cells, these compounds could prevent XRCC4/Lig4 interaction at the stage of *de novo* protein synthesis and would not disrupt preformed complexes. Therefore, it will be necessary to establish the optimal duration of cell treatment required to achieve a depletion of the nuclear stock of XRCC4/Lig4 complex sufficient to impair Lig4-dependent end-joining of DSBs.

In summary, we have isolated of the first compound able to target the C-terminal domain of Lig4 and prevent *in vitro* its interaction with XRCC4. Our results (i) validate the clamp domain of Lig4 as a minimal target for *in silico* screening strategy, (ii) support the feasibility of preventing the extremely high-affinity XRCC4/Lig4 interaction with small molecules *in vitro*, (iii) provide a model of Lig4 interaction with a moderate affinity compound that may now serve as a basis for the rational design of derivative molecules with better affinity for the Lig4 C-ter domain.

## Methods

### Proteins expression and purification

XRCC4 (1–336) was expressed in *Escherichia coli* BL21 (DE3) cells and purified by Ni-NTA affinity and Q Sepharose chromatography as described^49^,^50^. N-terminal poly-histidine tagged WT Lig4 C-ter domain (654–911) was expressed using expression pWY1119 plasmid in *Escherichia coli* BL21 (DE3) cells by growth in selective LB medium at 37 °C to OD_600 nm_ = 0.5. Cells were then maintained 1h at 15 °C and induced by addition of 0.5 mM ITPG and further incubation at 15 °C for 16 hours. Cells were harvested, lysed in Ni-A buffer (30 mM tris pH 8.0, 750 mM KCl, 10% glycerol, 10 mM imidazole, 0.2 mM ß-mercapthoethanol) complemented with 1 mg/mL Lysozyme, 1 mM PMSF, 0.5% NP-40 and protease inhibitors cocktail (Roche), clarified by high speed centrifugation and loaded on a HisTrap HP column. The column was washed extensively with Ni-A buffer and the proteins eluted with Ni-B buffer (Ni-A + 500 mM imidazole). The fractions were then diluted to 0.1 M KCl using 20 mM HEPES pH 7.5, 50 mM KCl, 10% glycerol, 2 mM EDTA, 1mM DTT and loaded on a HiTrap Q HP column. Protein was eluted using a linear KCl gradient from 0.05 to 0.5 M in 60 mM NDSB-195 containing tubes. The peak fractions containing the Lig4 C-ter domain were concentrated, aliquoted, flash frozen in liquid nitrogen and stored at −80 °C. Lig4 BRCT2 domain C-terminal GST tagged was expressed in *Escherichia coli* Rosetta cells by growth in selective LB medium at 37 °C to OD_600 nm_ = 0.5. Cells were then induced by addition of 0.5 mM ITPG and further incubation at 15 °C for 16 hours. Cells were harvested, lysed in binding buffer (50 mM tris pH 8.0, 500 mM NaCl, 10% glycerol, 1 mM DTT, 2.5 mM EDTA) complemented with 1 mg/mL Lysozyme, 1 mM PMSF, 0.5% Triton X-100 and protease inhibitors cocktail (Roche), clarified by high speed centrifugation and loaded on a GSTrap column. The column was washed extensively with binding buffer and the proteins eluted with elution buffer (binding buffer +20 mM reduced glutathione). Pure fractions were then pooled together and cleaved with TEV protease overnight at 4 °C. Cleaved protein was then injected into a Superdex 200 gel filtration column to remove the GST tag and in an NMR buffer (20 mM HEPES pH 7.5, 100 mM KCl, 2 mM EDTA, 1 mM DTT, 66 mM NDSB-195, D20 (10%), 10% glycerol). The peak fractions containing the pure Lig4 BRCT2 domain were concentrated, aliquoted and stored at −80 °C.

### Chemical compounds

According to Fig. S1, pyrimidinones 5 (#3091B) and 6 (#3101) were synthetized starting from propanone as previously reported^51^,^52^,^53^. The Strecker reaction (scheme detailed in Fig. S1) provided the aminonitrile 1, while subsequent conversion to the N-Cbz-protected intermediate 2 and hydroxylamine addition to the nitrile afforded amidoxime 3 as crystalline solid. The heterocyclic core was rapidly assembled by a two-component coupling reaction between amidoxime 3 and dimethyl acetylenedicarboxylate (DMAD). A thermal rearrangement of Michael adduct 4 afforded the key 5-hydroxypyrimidinone 5 (compound #3091B). Protection of 5-hydroxy group as benzoate ester leaded to 6 (compound #3101). Preparation of Methyltetraethyleneglycol 2-(1-{[(Benzyloxy)carbonyl]amino}-1-methylethyl)-5-(benzoyloxy)-6-oxo-1,6-dihydropyrimidine-4-carboxamide (compound **7**). First, Methyltetraethyleneglycol 2-(1-{[(Benzyloxy)carbonyl]amino}-1-methylethyl)-5-hydroxy-6-oxo-1,6-dihydropyrimidine-4-carboxamide was prepared as follows: A 10 mL flask was charged with 5 (30 mg, 0.083 mmol, 1 eq.) and methyltetraethyleneglycol amine (51 mg, 0.25 mmol, 3 eq.) in methanol (1 mL). After stirring at reflux for 12 h., 10 mL of EtOAc was added. The reaction mixture was washed first with 10 ml of an aqueous solution of HCl (5%), then with water (10 ml) and brine (10 ml). The organic layer was dried over MgSO4 and concentrated under vacuum. The residue was purified by flash chromatography (gradient, EtOAc/petroleum ether, 0–100% in 15 min.) to produce 36 mg, 81% as dense yellow oil. 1HNMR (CDCl3, 300 MHz) δ ppm: 1.65 (6H, s, Me), 3.38 (3H, s, -OMe), 3.51–3.67 (14H, m, CH2-O-CH2-CH2-O-), 3.96 (2H, bs, N-CH2) 5.02 (2H, s, -CH2Ar-), 5.82 (1H, bs, NH), 7.29 (5H, bs, Ar), 8.01 (1H, bs, NH), 10.76 (1H, bs, OH), 12.36 (1H, bs, NH). 13CNMR (DMSO-d6, 75 MHz) δ ppm: 168.99, 158.86, 155.12, 137.32, 128.79, 128.24, 128.02, 127.56, 126.19, 71.75, 70.26, 70.07, 68.90, 65.76, 58.49, 55.39, 26.51. ES-HRMS (positive mode) calcd for C25H37N4O9 [M+ H]+: 537.2561. Found: 537.2559; [M+ Na]+: 559.2380. Then, to a stirred solution of Methyltetraethyleneglycol 2-(1-{[(Benzyloxy)carbonyl]amino}-1-methylethyl)-5-hydroxy-6-oxo-1,6-dihydropyrimidine-4-carboxamide (20 mg, 0.037 mmol, 1 eq.) in pyridine (1 mL), benzoic anhydride (100 mg, 0.442 mmol, 12 eq.) was added at room temperature. After stirring for 12 h. 10 ml of EtOAc was added. The reaction mixture was washed first with 10 ml of an aqueous solution of HCl (5%), then with water (10 ml) and brine (10 ml). The organic layer was dried over MgSO4 and concentrated under vacuum. The residue was purified by semi-preparative HPLC to produce 7 (10 mg, 41%) as dense yellow oil. 7: 1HNMR (CDCl3, 300 MHz) δ ppm: 1.51 (6H, s, Me), 3.36 (3H, s, -OMe), 3.45–3.65 (14H, m, -O-CH2-CH2-O-), 3.74 (2H, t, J = 5.4 Hz, NH-CH2), 5.05 (2H, s, -CH2Ar-), 5.34 (1H, bs), 7.34 (5H, m, Ar), 7.54 (2H, t, J = 7.5 Hz, Ar), 7.68 (1H, bs, Ar), 8.15 (2H, d, J = 7.2 Hz, Ar). 13CNMR (DMSO-d6, 75 MHz) δ ppm: 168.99, 162.25, 158.86, 155.12, 142.54, 137.32, 133.90, 130.31, 128.79, 128.61, 128.24, 128.02, 127.56, 126.19, 71.75, 70.26, 70.07, 68.90, 65.76, 59.39, 55.39, 26.51. ES-HRMS (positive mode) calcd for C32H41N4O10 [M+ H]+: 641.2823. Found: 641.2828; [M+ Na]+: 663.2647. The synthesis of methylated pyrimidinone (compound 8) has been previously described^51^,^52^,^53^ and the characterization data obtained in this work were in accordance with those of literature.

Raltegravir was purchased from Euromedex (Souffelweyersheim, France).

Compounds #231 and #36972 were purchased from Enamine (Riga, Latvia).

### Differential Scanning Fluorimetry

DSF experiments were performed into a white/clear 96-well PCR plate (Bio-Rad) in a 20 μL final volume to assess proteins thermostability through the unfolding temperature (Tm). The C-ter Lig4 protein was tested alone (with 2% DMSO or 2 mM of compounds) or in equimolar conditions with XRCC4 at a concentration of 5 μM in 20 mM HEPES pH 7.5, 100 mM KCl, 2 mM EDTA, 1 mM DTT, 66 mM NDSB-195, 10% glycerol and with 10X SYPRO Orange (Invitrogen) as dye. The purity of each protein sample was checked by a 10% polyacrylamide gel electrophoresis in denaturing conditions. PCR plates were sealed with optical quality sealing tape (Bio-Rad). Measurements were done using a CFX96 real-time PCR system (Bio-Rad) set to use the 480/500 excitation and 560/580 emission filters. Samples were heated from 20 to 89 °C. Each fluorescence measurement was taken every 0.3 °C/tps. Data were analyzed using the CFX Manager Software (Bio-Rad Laboratories, USA) and the Tm value estimated from the transition midpoint of the fluorescence curve, which corresponds to the temperature at which half of the protein population is unfolded.

### High-Throughput Docking

A screening campaign by high-throughput docking was performed using AutoDockTools v1.5.4 and Autodock Vina^34^. 11 MD-extracted clamp conformation structures of the Lig4 (residues 771–803) were used as targets with the coordinate pdbqt file prepared with AutoDockTools v1.5.4. During target preparation polar hydrogens were added and partial charges calculated according to the Kollman method^54^. The grid had a 40 Angstroms size in the x, y and z dimensions. A spacing of 1 Angstrom was used with the center of the grid placed at the center of gravity of the protein. During the docking, the side chains of the clamp were kept as rigid. An ensemble of ZINC database^32^ and in-house molecules was used. Raltegravir, a potent selective orally bioavailable HIV-integrase inhibitor is based on the dihydroxypyrimidine scaffold. This scaffold, and also the carboxamide derived compounds are characterized by improved drug like qualities^51^. Thus, in-house series of molecules of hydroxypyrimidinone family were included in the chemical database. Molecules were converted via Open Babel 2.2.3 into pdbqt files with added hydrogens and proper charge (at pH7.2) and 3D coordinates. The screening library has been filtered taking into account the ADMET properties of the compounds. Reactive compounds (covalent binders), warheads (non-covalent binders) as well as the PAINS compounds have been eliminated^55^,^56^,^57^, and the rest of the library has been filtered for druglikeness^58^,^59^. The initial part of the work was done using the Screening Assistant v.2 (sa2.sourceforge.net). Marvin and Calculator Plugins were used for drawing and displaying chemical structures, as well as for structure property prediction and calculation (Marvin 6.1.5, 2013, ChemAxon, www.chemaxon.com). The OpenEye software was used to create tautomers, select molecules with protonation states appropriate for the physiological pH and to generate rotamers for subsequent docking (OEDocking, version 3.0.1, OpenEye Scientific Software, Inc., Santa Fe, NM, USA, www.eyesopen.com, 2013). Docking was performed first with Vina, then with FRED^60^. The final filtered database contained around ~95.000 molecules. Docking was performed on eight CPUs with an exhaustiveness value of 80 and a maximum output of 20 conformers. Ligands from the rigid docking were ranked according to the Vina score and a threshold value of −7 kcal/mol was chosen to select the most promising hits. From this, 29 molecules were selected for a MD simulation step and these top compounds were selected based on the criteria of number of non-polar and H-bond contacts inside the clamp structure. MD simulations of 10ns for each 29 C-ter Lig4/molecule complexes were performed to determine the stability over time (RMSD) of the interaction, persistent contacts for each molecule inside the clamp domain as well as details on the molecule binding mode and energy. The binding energy was calculated from the last 4ns of simulation to rank each candidate and with the generalized-born method^61^.

### Molecular Dynamic simulations

MD simulation was done on the XRCC4/C-ter Lig4 protein complex (PDB entry 3II6, residues 654–911) for 10 ns. It was also done with each 29 C-ter Lig4/molecule complexes for the same calculation time. These simulations were performed with the Amber 9 software (1–3) including the all-atom ff03 force field for protein parameterization and the generalized AMBER force field (GAFF) for ligands. The full systems were protonated and centered in a periodic TIP3P water box model, containing 37 Na+ ions and 61875 water molecules. The equilibration of the system was achieved in several steps. Initially, the energy of the system was minimized by 1000 cycles of the steepest descent (SD) algorithm, with the solute held fixed, by constraining its Cartesian coordinates using a harmonic potential with the force constant k equal to 100 kcal/mol/Å^2^. In the second step, the energy was minimized by 500 cycles of SD and 1500 cycles of the conjugate gradient (CG) algorithm, with weakly restrained solute (k = 10 kcal/mol/Å^2^). Next, a short 20 ps MD run was performed on weakly restrained solute with temperature varying linearly from 0 to 300 K. The temperature control was achieved using the Langevin dynamics with the collision frequency parameter γ equal to 1.0 ps^−1^. The integration step used in this run was 1 fs. Throughout the calculations a cutoff of 12 Å was used for electrostatic interactions. The MD simulation continued for 100 ps at constant temperature at 300 K with no restraints, with the integration step of 2 fs. Finally, a 10 ns run with constant pressure of 1 bar was launched, with atomic coordinates saved every 10 ps. The Langevin dynamics was used to control the temperature, with γ = 1.0 ps^−1^, while the pressure was controlled by the anisotropic Berendsen barostat with the pressure relaxation time τ_p_ = 2 ps. Bonds involving hydrogen were constrained with the SHAKE algorithm^62^. The calculation was performed in parallel on two PowerEdge R410 servers, each equipped with two quadricore Intel Xeon X3430 processors.

### STD-NMR and TR-NOESY

For STD-NMR spectra, protein buffer was exchanged against an NMR buffer with 20 mM HEPES pH 7.5, 100 mM KCl, 2 mM EDTA, 1 mM DTT, 66 mM NDSB-195, D_2_0 (10%), 10% glycerol using two cycles of 10-fold dilution, followed by concentration on Amicon Ultra (regenerated cellulose 3kDa, Millipore, Ireland) at 4000 g for 60 min. NMR tubes were prepared in 3 mm capillaries (165 μL) containing 14 μM of C-ter Lig4 protein and 1mM of ligands from 100% DMSO-d_6_ stock solutions. On-resonance irradiation of the protein was set to −600.13 Hz and off-resonance at 18000 Hz. The selective irradiation was performed with a gaussian pulse set at 86 Hz with a 50 ms length. NMR spectra were recorded at 283 K (10 °C) on a Bruker Avance 600 spectrometer equipped with a TXI cryoprobe. NMR data were processed and analyzed using Topspin 3.2 NMR software (Bruker). For TR-NOESY spectra, protein buffer was the same as for STD-NMR experiments. Samples were prepared with 165 μL in 3 mm capillaries containing 1 mM of ligands from 100% DMSO d_6_ stock solutions and with or without 14 μM of C-ter Lig4 protein. Experiments were measured with 8 scans and a mixing time of 200 ms. NMR spectra were recorded at 298 K (25 °C) using a Bruker Avance 600 spectrometer equipped with a TXI cryoprobe. NMR data were processed and analyzed using Topspin 3.2 NMR software (Bruker).

### Native gel-based assays for protein-protein interaction

10 pmol of purified recombinant C-ter Lig4 protein was combined with an increasing amount of each tested molecule at room temperature during 15 min. The final DMSO concentration was maintained constant for each sample. Then, 10 pmol purified recombinant XRCC4 protein was added in the mix with the reaction buffer (20 mM Tris pH 8.0, 50 mM KCl, 0.1 mM DTT and 5% glycerol) in 10 μl final reaction volume (the final concentration of each protein was 10 μM). The reaction was incubated during 15 min at room temperature. Reactions were loaded on a 6% native PAGE in TBE and run at 4 °C (with a 30 minutes pre-running of the empty gel). The gel was colored with Instant Blue (Expedeon) and image was captured with an Epson Perfection 2480 Photo scanner. Quantitative analysis of the gel was performed with the ImageJ software (version 1.4).

## Additional Information

**How to cite this article**: Menchon, G. *et al.* Structure-Based Virtual Ligand Screening on the XRCC4/DNA Ligase IV Interface. *Sci. Rep.* **6**, 22878; doi: 10.1038/srep22878 (2016).

## Supplementary Material

Supplementary Information

## Figures and Tables

**Table 1:**
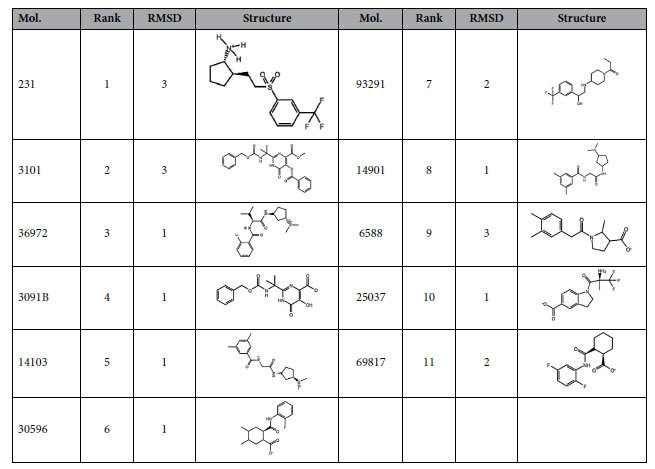
Best compounds interacting with the Lig4 C-ter domain selected by MD simulation.

11 selected compounds making persistent contacts within the clamp domain of the C-ter Lig4 protein after a 10ns MD simulation. Molecules were ranked according to their predicted binding energies. According to the stability of each molecule in the Lig4 clamp domain, a RMSD score was qualitatively set to 3, 2 or 1 for a very stable molecule, a fluctuating molecule with a unique binding mode or a very fluctuating molecule and different binding modes, respectively (see also Fig. 3A).

**Table 2:** Results of analysis by DSF and STD -NMR spectroscopy of the interaction of selected compounds with the Lig4-Cter domain.

Compounds	Tm ( °C)	STD-NMR signal
DMSO	38.2 ± 0.2	N.D
231	37.5 ± 0.2	+
3101	36.2 ± 0.1	+
36972	36.9 ± 0.2	−
3091B	38.2 ± 0.2	−
