# Supplementary material for: Structure-Based Virtual Ligand Screening on the XRCC4/DNA Ligase IV Interface
Source: Sci Rep. 2016 Mar 11;6:22878. doi: 10.1038/srep22878 (PMC4786802; doi:10.1038/srep22878)

**Structure-Based Virtual Ligand Screening on the XRCC4/DNA Ligase IV Interface**

Grégory Menchon^1,2^, Oriane Bombarde^1,2^, Mansi Trivedi^1^, Aurélie Négrel^4,5,6,7#^, Cyril Inard ^3^, Brigitte Giudetti^3^, Michel Baltas^3^, Alain Milon^1^, Mauro Modesti^4,5,6,7^, Georges Czaplicki^1*^, Patrick Calsou^1,2*^

^1^ Institut de Pharmacologie et de Biologie Structurale, Université de Toulouse, CNRS, UPS, France

^2^ Equipe labellisée Ligue Nationale Contre le Cancer

^3^ Synthèse et physico-chimie de molécules d'intérêt biologique, Université de Toulouse, CNRS, France

^4^ Centre de Recherche en Cancérologie de Marseille, CNRS, UMR7258, Marseille, F-13009, France;

**^5^** INSERM, U1068, Marseille, F-13009, France;

**^6^** Institut Paoli-Calmettes, Marseille, F-13009, France;

**^7^** Aix-Marseille Université, Marseille, F-13284, France;

* To whom correspondence should be addressed: Patrick Calsou, IPBS (Institut de Pharmacologie et de Biologie Structurale); BP 64182, 205 route de Narbonne, F-31077 Toulouse, Cedex4, France, Tel: +33 561 175 970, Email: [patrick.calsou@ipbs.fr](mailto:patrick.calsou@ipbs.fr) ; Georges Czaplicki, IPBS (Institut de Pharmacologie et de Biologie Structurale); BP 64182, 205 route de Narbonne, F-31077 Toulouse, Cedex4, France, Tel +33 561 175 404, Email : [georges.czaplicki@ipbs.fr](mailto:Georges.Czaplicki@ipbs.fr)

# present address : Sigma Aldrich, 80 rue de Luzais, 38297 Saint Quentin Fallavier

**Supplementary figure legends.**

**Supplementary Figure S1. Synthesis scheme of compound #3101 and derivatives.** Reagents and conditions: (a) NH_4_Cl, 28% NH_3_, NaCN, EtOH, H_2_O, 25-50°C, 4 days; (b) Cbz-Cl, Na_2_CO_3_ , H_2_O, 0°C- rt, 5 h; (c) 50% NH_2_OH - H_2_O, iPrOH, 60°C, 5 h; (d) DMAD, MeOH, rt, 4 h; (e) Xylene, 150°C, 48 h; (f) (PhCO)_2_O, Py, rt, 12-15 h; (g), (h), see experimental procedures.

**Supplementary Figure S2. Analysis of the interaction of compound #3101 with the Lig4 BRCT2 and the full Lig4-Cter domains by Saturation Transfer Difference - NMR spectroscopy.**

**A/** Analyis by DSF of the thermal stability of the Lig4 BRCT2 domain. Unfolding is followed with 10X SYPRO Orange dye and between 20 and 89°C. Tm values are estimated from the transition midpoint of the fluorescence curve. From left to right: picture of BRCT2 domain; DSF profile and BRCT2 band on SDS-PAGE. **B/** One-dimensional ^1^H STD-NMR spectra of the molecule #3101 with the Lig4 BRCT2 domain control (a) and in association with the Lig4 C-ter fragment (b). Protons making close contacts with the protein show significant resonance intensity enhancements. Due to baseline distortion and buffer, only the aromatic regions are represented.

**Supplementary Figure S3 : Full-size exposure of native gel data.** Panels A, B, C and D correspond to the original scans of gels presented in Fig. 5 A, B, C and D, respectively, with identical numbering of the wells between original and cropped gels.


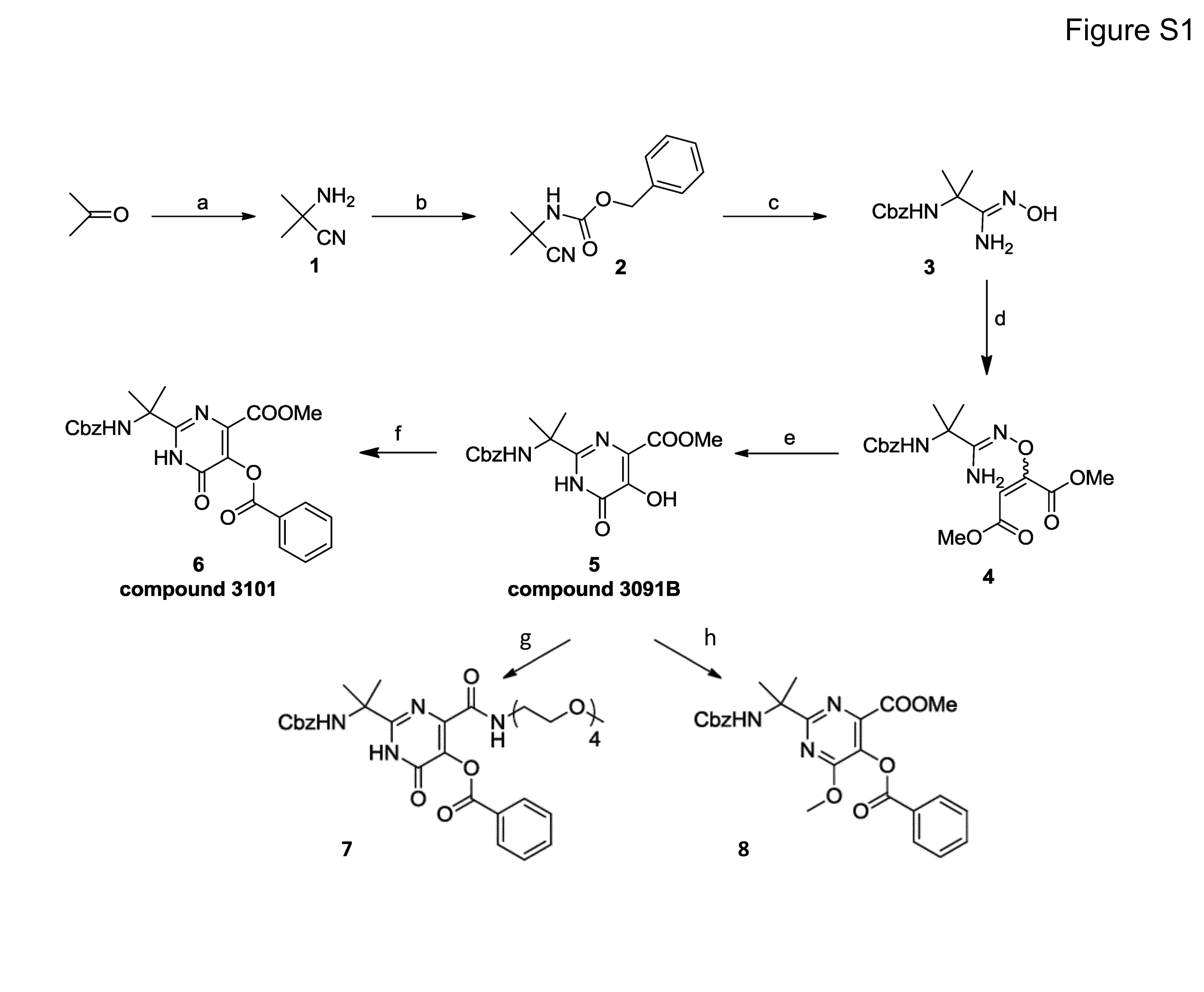


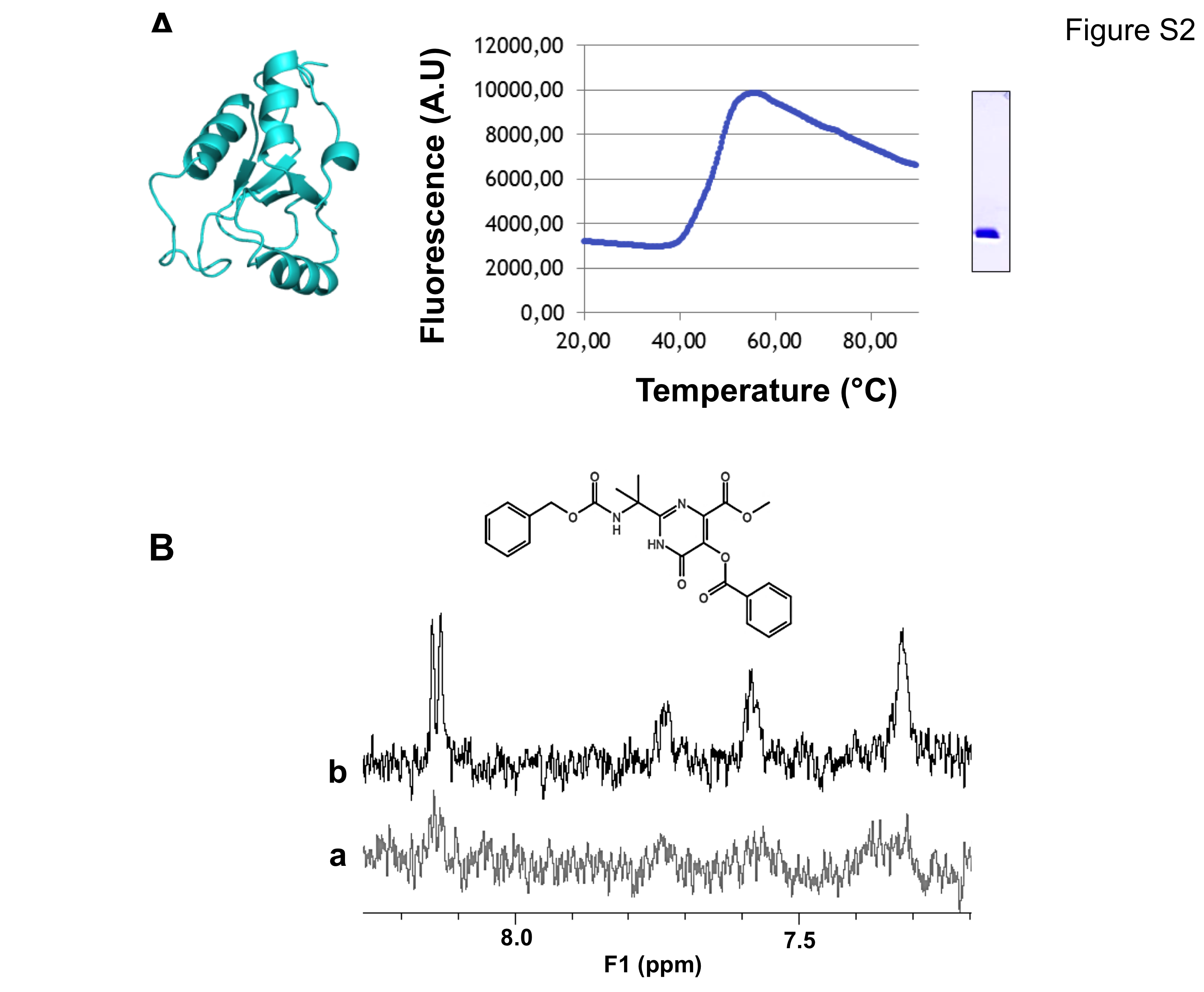


Figure S3


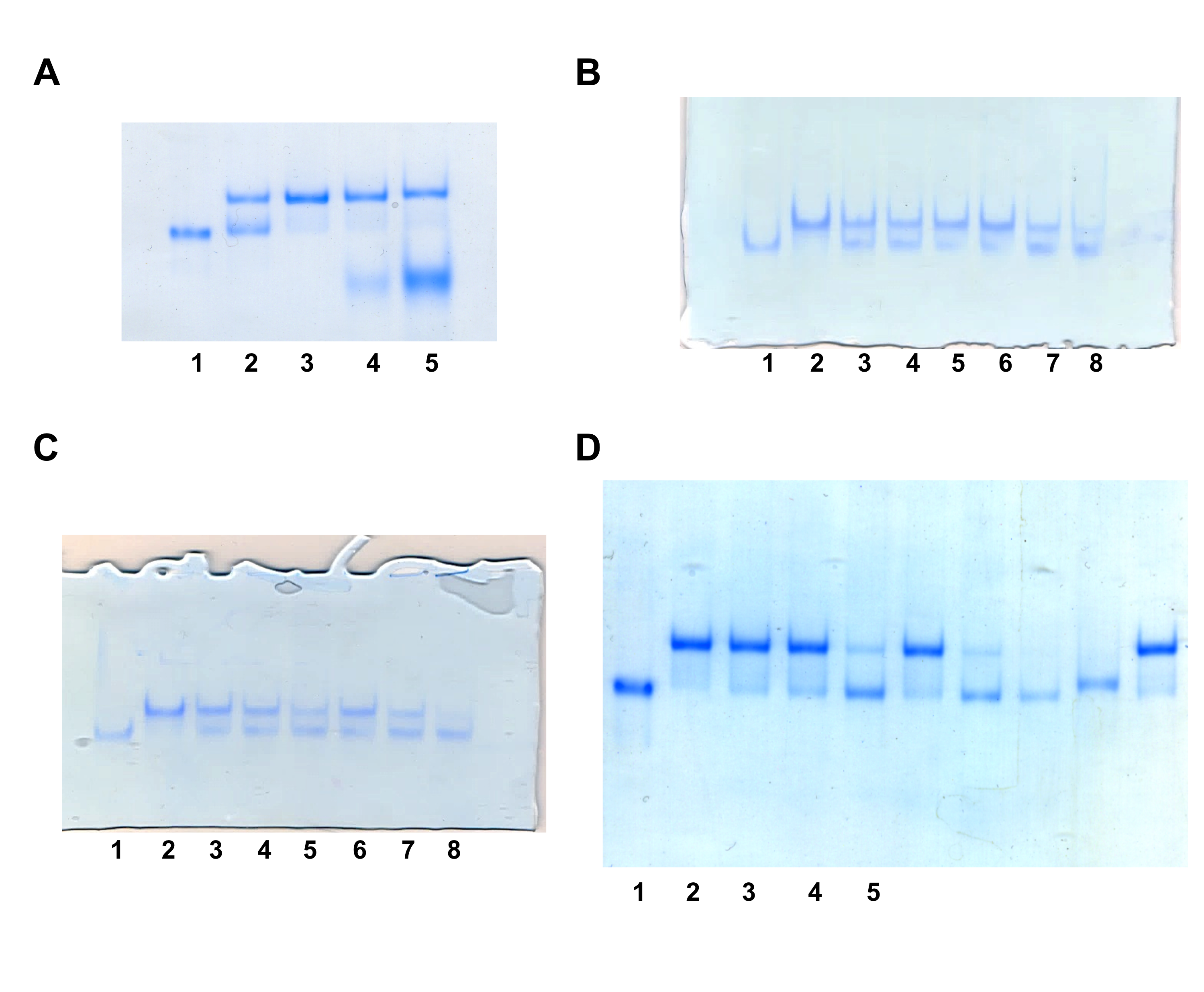

Supplement: Supplementary Information [file srep22878-s1.doc]
